# Strategies Toward Synthesis and Conversion of Lignin Model Compounds

**DOI:** 10.1002/chem.202500805

**Published:** 2025-06-11

**Authors:** Gary N. Sheldrake, Christopher W. J. Murnaghan

**Affiliations:** ^1^ School of Chemistry and Chemical Engineering Queen's University Belfast David Keir Building, Stranmillis Road Belfast BT9 5AG UK; ^2^ Department of Biology Edge Hill University St Helen's Road Ormskirk L39 4QP UK

**Keywords:** lignin, organic, sustainable

## Abstract

The push toward a renewable society where the chemicals being used on a daily basis come from sources which are not going to be depleted within the next few decades is highly sought after. Biomass is one of the most promising opportunities to establish self‐sustainability for the human race. This review article takes a look at some of the key methods which have been employed for the synthesis of important lignin model compounds, and the synthetic techniques are discussed throughout the first section. The second section of this review is focused on some of the major strategies for the conversion of lignin model compounds throughout the literature. This review serves as a good starting point for someone who is relatively new to the field of lignin model synthesis and valorization.

## Graphical abstract

The conversion of lignin toward value‐added products relies on the cleavage of C─C and C─O bonding patterns, which has proven to be a difficult task previously, with numerous factors at play. The employment of model compounds which are fashioned on the structure and linkages found within lignin aids in the development of catalytic processes which can then be applied for the scission of linkages within native lignin. This review focuses on the strategies which have been used for the synthesis and conversion of key model compounds of lignin and the products obtained following their breakdown studies.

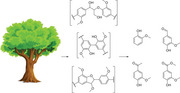

## 1 Introduction

Due to the ever‐expanding global population, the need for resources which have sustainable roots is at an all‐time high,^[^ ^1^ ^]^ and as such, more methods must be developed in order to meet the demand. The time has come for there to be more advances in the production of fuels from renewables as opposed to fossil fuels, as this method of obtaining these is finite.^[^ ^2^ ^]^ Organic chemistry relies heavily on the use of solvents for fine chemical synthesis and also relies on heat for some transformations, all produced from fossil fuels. Therefore, it is imperative not only for the future of organic chemistry but also, the prosperous growth of humanity that renewable methods are found to extract organic materials in order to replace oil. Oil is the remains of life under the earth's crust, which has been exposed to extreme pressure due to the difference in porosity between shale and sandstone. Therefore, it would make sense to turn to a source that is in abundance and has the potential to be replaced once consumed. The use of trees as a source of energy^[^ ^3^ ^]^ has long been established, but can be a very wasteful resource, as the energy obtained is usually from combustion alone.

However, research into obtaining more products of value from tree bark has been prolific in this century,^[^ ^4^, ^5^, ^6^ ^]^ with special attention focusing on the biochemical polymers in wood. Over the years, an abundance of work in this field has been performed to attempt to extract the chemical constituents of importance out of tree matter and break them down into smaller blocks. This valorization provides potential for new applications such as: fine chemicals for synthesis, including solvents, and also renewable fuel sources. Considering the depolymerization of cellulose, the products obtainable from this polymer include bioethanol, biobutanol, HMF, levulinic acid, and anhydrosugars like levoglucosenone.^[^ ^7^ ^]^

**Figure 1:**
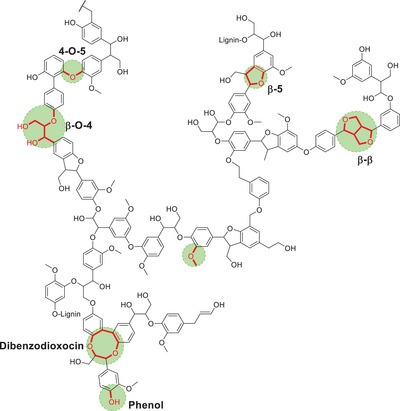
Representative structure of lignin showing the main functional groups and linkages.^[^ ^1^ ^]^

**Figure 2:**
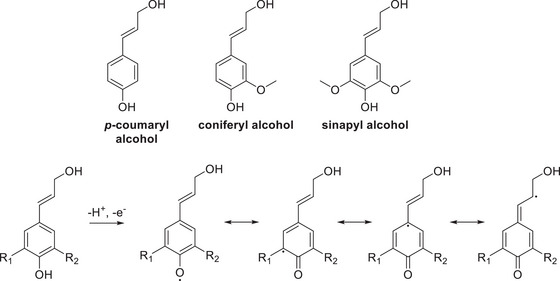
The three main monomer precursors and their resonance forms of the radical in the main precursors.^[^ ^8^, ^9^ ^]^

**Figure 3:**
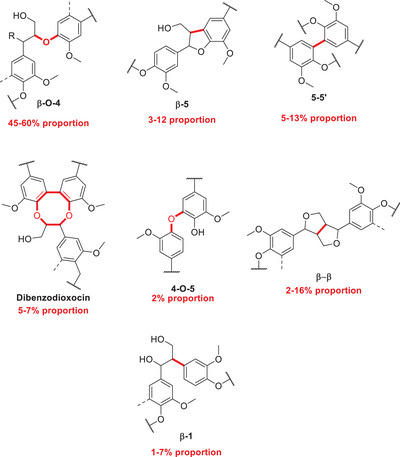
The different types and proportions (in both softwood and hardwood) of linkages, which are found in lignin formation of β‐β, β‐5, and β‐O‐4 linkages from the combination of the radical species.^[^ ^11^ ^]^

**Figure 4:**
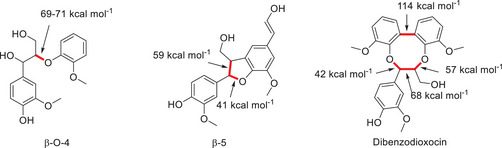
Selection of linkages found within lignin with their associated bond enthalpies.^[^ ^14^ ^]^

The three main monomer precursors of lignin, or monolignols, have been found to be coniferyl alcohol, *p*‐coumaryl alcohol and sinapyl alcohol. The proportion of each monolignol residue in the lignin polymer determines its properties, i.e., hardwoods or angiosperms have a relatively high proportion of sinapyl units to develop syringyl polymers, whereas softwoods or gymnosperms are composed mainly of coniferyl alcohol units to develop guaiacyl polymers.^[^ ^10^ ^]^ As previously mentioned, the linkages in the lignin structure are of extreme importance as these are the targets in numerous processes in order to extract chemicals of interest and value (Figure 1, 2, 3, 4, 7, and 8).

**Figure 5:**
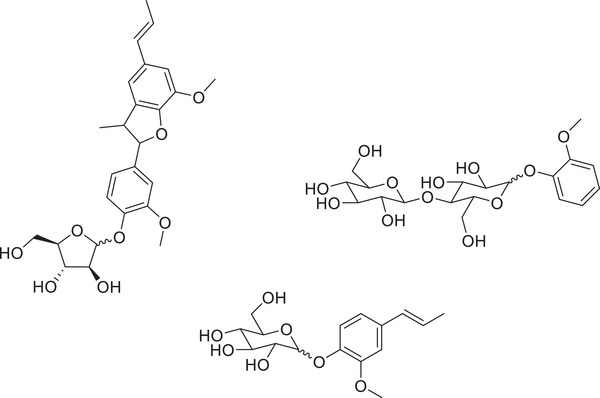
Range of phenyl glycosidic class of lignin‐carbohydrate complex model compound, which was synthesized by Sheldrake et al.^[^ ^51^ ^]^

The coupling reactions of the monolignols to form lignin are mainly radical, similar to the case of the formation of the highly reactive quinine methide intermediate,^[^ ^8^ ^]^ as exemplified by the resonance structures shown below. The generation of the reactive species is the formation of the aroxyl radical, which is produced by the action of the peroxidase enzyme on the hydroxycinnamyl alcohol. DFT calculations performed^[^ ^12^ ^]^ by Smith et al. found that for the coupling reactions, they are all favourable in terms of ΔG; however, the couplings to produce the 4‐O‐5 and the 5‐5 linkages are only weakly favorable. Smith et al. assert that the reason for this is due to the likelihood of reversibility due to hydration and tautomerization is high. It was noted by Sankar^[^ ^13^ ^]^ that the varying proportions of each linkage found in lignin differ across species. According to Sankar et al., the lowest proportion of all linkages is the β‐β, whereas the most abundant linkage is the β‐O‐4. Considering the plethora of bonds which are found within lignin in Figure 5, the diversity of linkages results in an overall “tough nut to crack” when it comes to finding a catalytic system which has selectivity toward certain bonds over others.

### 1.1 Model Compounds and Linkages

As the exact structure of lignin is poorly understood, the complex and intact polymer is difficult to isolate. A good approach to further the understanding of lignin is by synthesizing model compounds, which contain one or more of the same linkages found in the native structure. The breadth of literature when it comes to lignin model compounds is vast, and there have been numerous successful efforts toward the synthesis of each of the linkages found in lignin. The most common linkage which has been synthesized in the literature is the β‐O‐4 linkage because it is the most common bond found in native lignin and because of its ease of installation in model compounds. Much work has been done using these models as they provide a reliable, robust set of results, typically as they are used as the benchmark within the literature.

### 1.2 Dimeric Model Compounds

The simplest of all the model compounds of lignin are those which consist of two aryl rings linked by one of the specific linkages which are found in native lignin. Lancefield and Westwood directed their efforts into synthesizing the β‐β, β‐O‐4, and the β‐5 linkages in dimeric form. The simplest of all the model compounds of lignin are those which consist of two aryl rings linked by one of the specific linkages which are found in native lignin. Lancefield and Westwood^[^ ^15^ ^]^ directed their efforts to synthesizing the β‐β, β‐O‐4, and β‐5 linkages in dimeric form (Scheme 1, 2, 3, 4, 5, 6, 7, 8, 9, 10, 11, 12, 13, 14, 15, 16, 17).

**Scheme 1:**
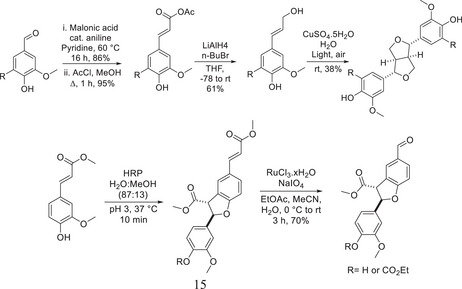
Synthesis of the β‐β and β‐5 dimer by Westwood.^[^ ^15^ ^]^

**Figure 6:**
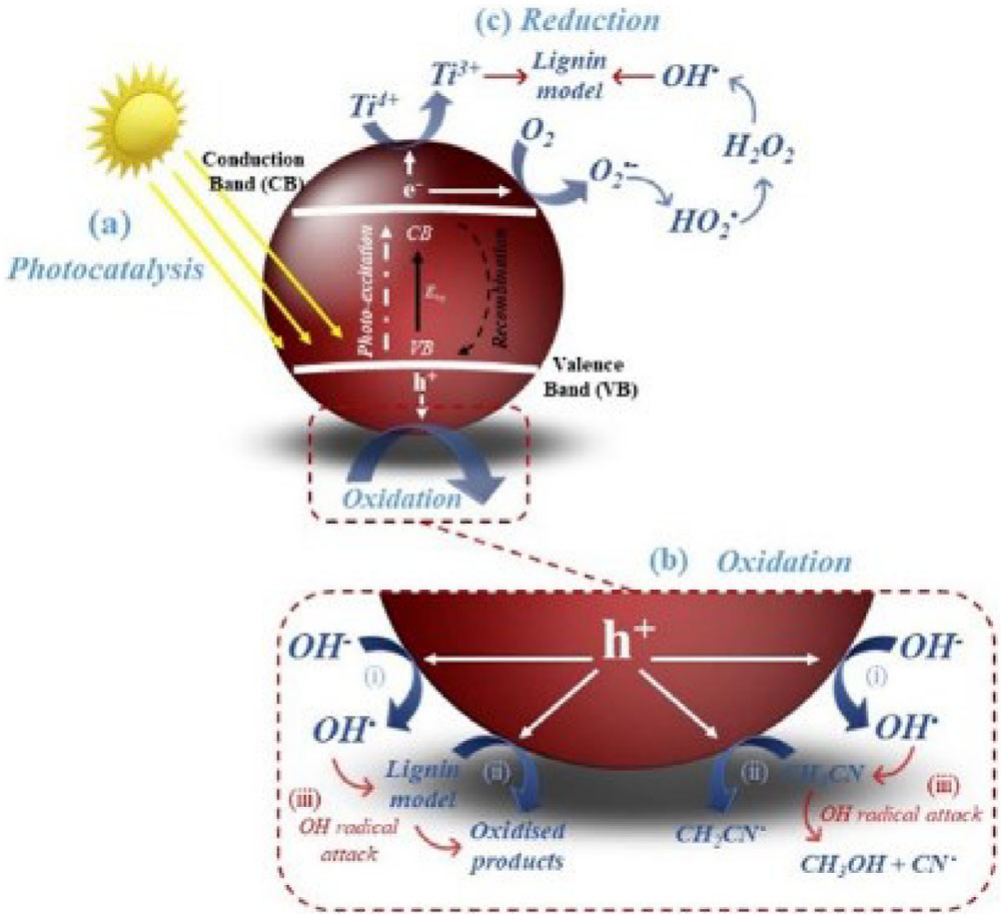
Overview of the potential photocatalytic degradation of lignin models which shows a) the photocatalysis mechanism; b) oxidation reactions at the valence band including (1) OH• formation, (2) direct hole oxidation, and (3) OH• attack of lignin models and CH_3_CN; c) the formation of O2^•−^ at the conduction band and OH• generation via H_2_O_2_ fission adapted from Murnaghan et al.^[^ ^79^ ^]^

The synthesis of this resinol‐type dimer was effected by extension of the aldehyde functionality of syringaldehyde. Reduction of this ester provided the alcohol, dimerization then provided the β‐β species. Following the synthesis of the β‐β dimer, Westwood et al. also synthesized the β‐5 dimer, by starting with the dimerization of methyl ferulate followed by oxidative cleavage of the alkenoic acid to the aldehyde. The implementation of the three linkages between the two model compounds was indeed a very important contribution in the fact that it is one of the few examples of synthetic methodology toward both the β‐β and β‐5 linkages. The furnishing of the stereochemistry is a bonus, considering the *anti* configuration in the benzofuran ring. There have not been many examples of the synthesis of both the β‐β and β‐5 linkages within the literature; however, those that have been reported are typically done so using an oxidative dimerization methodology like Tran et al.^[^ ^16^ ^]^ in the synthesis of the β‐β model Eudesmin^[^ ^17^ ^]^ or the work of Sheldrake et al.^[^ ^18^ ^]^ where a dimeric β‐5 linkage was synthesized as an intermediate in the synthesis of a hexameric model Figure 6. Also, a notable mention is the work of Fang et al.,^[^ ^19^ ^]^ who, in one synthetic route, developed methodology based on the initial work of Lundquist^[^ ^20^ ^]^ to synthesize both the β‐1 and subsequently the β‐5 through an acid‐catalyzed ring closure reaction.

**Scheme 2:**
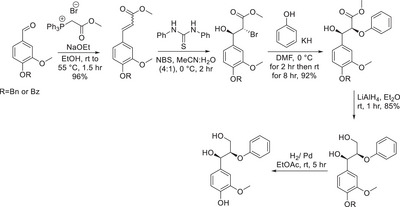
Use of Wittig chemistry in the β‐O‐4 synthesis by Mukhtar.^[^ ^27^ ^]^

With the β‐O‐4 linkage being the most common across both hardwood and softwood species, there have been many examples of the synthesis of this type of linkage in the literature, with the majority centering around the formation of the guaiacylglycerol‐β‐guaiacyl ether as a lignin model.^[^ ^21^, ^22^, ^23^, ^24^, ^25^, ^26^ ^]^ An interesting synthesis of the β‐O‐4 model linkage in a dimer, depicted below in Scheme 2, was published by Mukhtar et al.^[^ ^27^ ^]^ where the formation of the bromohydrin was the preceding step in the formation of the ether linkage.

**Scheme 3:**
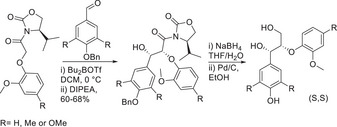
Synthetic route toward the diastereomeric β‐O‐4 dimer by Hartwig et al.^[^ ^30^ ^]^

**Scheme 4:**
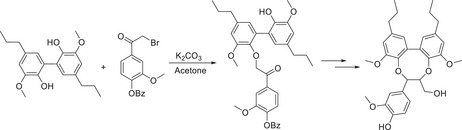
Overall transformation performed by Brunow et al.^[^ ^34^, ^35^, ^37^ ^]^

**Scheme 5:**
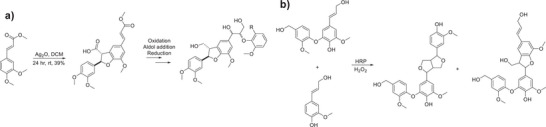
a) Synthesis of the trimer containing both the β‐O‐4 and the β‐5 linkages by Barta^[^ ^40^ ^]^ and b) Yue's enzymatic synthesis of the β‐β and the β‐5 linkages in the 4‐O‐5 models.^[^ ^41^ ^]^

**Scheme 6:**
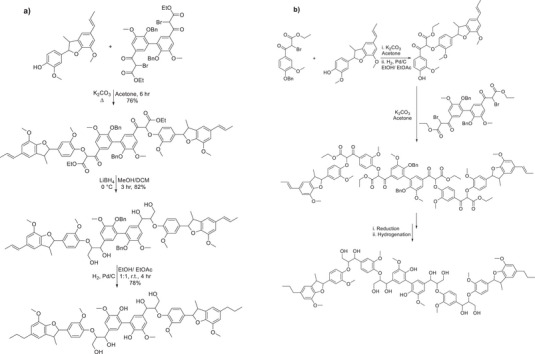
a,b) Synthetic routes devised for the formation of hexameric (a) and octameric (b) lignin model compounds containing β‐O‐4, β‐5, and 5‐5′ linkages.

**Scheme 7:**
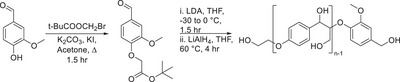
Synthesis of the polymeric model containing the β‐O‐4 linkage by Katahira.^[^ ^42^ ^]^

**Scheme 8:**
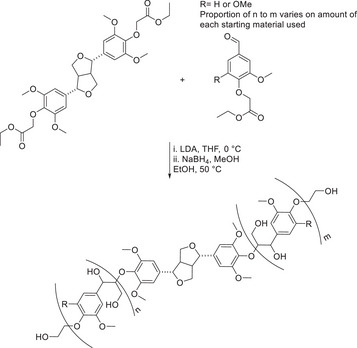
Synthesis of the polymeric model containing both β‐β and the β‐O‐4 linkages by Westwood.^[^ ^15^ ^]^

Recent efforts have also focused on synthesizing the β‐O‐4 dimer.^[^ ^28^, ^29^ ^]^ Hartwig et al.^[^ ^30^ ^]^ were able to produce both the (*R,R*) and (*S,S*) via installation of an Evans auxiliary aldol reaction and then the diastereoisomers producing the (*R,S*) and (*S,R*) by stereoinversion.

Another important contribution to the field of enantio and diastereomeric model compounds was put forward by Hishiyama et al where the use of a different chiral sultam auxiliary enabled a pathway toward dimeric β‐O‐4 models with mixtures of erythro and threo stereochemistry.^[^ ^31^ ^]^ There are very few reports of such enantiomeric selectivity when applied to lignin model synthesis.^[^ ^32^ ^]^ This may be due to the fact that within nature lignin is composed in a nonselective fashion, and indeed it is likely that there will be some random assembly of fragments giving rise to diastereomers. With regard to the implementation of strategies which can affect or retain the isomers present within lignin model compounds, Bolm et al.^[^ ^33^ ^]^ compared the reactivity of ball‐milling versus enzyme for the selective esterification of the γ position in a β‐O‐4 model compound.

### 1.3 Trimeric Model Compounds

The synthetic methods which have been developed and implemented for the formation of model compounds which themselves contain three aromatic rings and thus are termed “trimeric” have been reported, and largely (again) the presence of β‐O‐4 is at the forefront of the content. There are a few notable examples of the syntheses of the dibenzodioxocin linkages by Karhunen et al.,^[^ ^34^, ^35^ ^]^ which is a feat of synthetic triumph considering the arrangement and twist boat chair the structure adopts.^[^ ^36^ ^]^ The synthetic route, which was devised for the synthesis of this cyclic trimer, begins with the dimerization of dihydroisoeugenol to furnish a 5‐5‘ linkage with K_3_[Fe(CN)_6_], which was then reacted with a bromoketone to yield an ether linkage. The key cyclization step came in the form of a reaction of a benzylic alcohol functionality with TMSBr and shaking with NaHCO_3_ to provide the eight‐membered ring.

The initial work by Hartwig in the synthesis of the diastereomeric model dimers laid the foundation also for the synthesis of trimeric model^[^ ^30^ ^]^ compounds, which also bear stereogenic carbons. In a sense, this is an impressive piece of synthetic work through the use of chiral auxiliaries; however, the random fashion in which linkages are formed in nature means that there is no discrimination in terms of chirality. In terms of other important contributions to the trimeric catalogue of lignin models, the β‐O‐4 linkages synthesized by Lahtinen^[^ ^38^ ^]^ and Brandi,^[^ ^39^ ^]^ the latter of which was an important contribution in the formation of a higher lignin model oligomer. In terms of structural diversity, the synthesis of a timeric species which contains both β‐O‐4 and β‐5 linkages was reported by Barta et al., in which the oxidative dimerization in the presence of Ag_2_O furnished the benzofuran β‐5 linkage.

### 1.4 Higher Oligomeric Models

In order to more accurately reflect how lignin is found in nature, the synthesis of more representative model compounds which contain more than one type of linkage and also a larger number of aromatic rings. Significant work by Sheldrake et al.^[^ ^18^ ^]^ where a hexameric and octameric lignin model compound was synthesized using “robust” chemistry from first principles. The use of an oxidative dimerization pathway for the formation of both the dehydrodiisoeugenol and the 5‐5′ linked diacetovanillone species was instrumental in furnishing the backbone of both hexamer and octamer structures.

Interesting work by Katahira et al.^[^ ^42^ ^]^ demonstrated the synthesis of polymeric β‐O‐4 species in a simple three‐step procedure. The installation of an ester in vanillin allowed for the Aldol C─C bond forming reaction to implement the β‐O‐4 linkage, followed by reduction of all carbonyl and ester functionalities. This is not the first example of the synthesis of an oligomeric β‐O‐4 lignin model compound through similar chemistry.^[^ ^43^, ^44^, ^45^ ^]^

An oligomeric lignin model compound containing β‐O‐4 and β‐β linkages was synthesized by Westwood et al.^[^ ^15^ ^]^ the use of aldol chemistry unlocked the route toward this polymeric model compound. The reduction of the esters present in the polymer with borohydride led to the final deprotected lignin model compound.

With reference specifically to the synthesis of the higher oligomeric model compounds, these represent the more complex types of models available within the literature. However, with respect to the work by Westwood and Katahira via the use of repeating Aldol chemistry, these models are limited by the linkage type present. The oligomers which have been reported by Westwood and Katahira are extremely beneficial in the fact that their potential enormity mimics lignin, which has been extracted from biomass. In contrast, the hexameric and octameric lignin models reported by Sheldrake, although much smaller in molecular weight, do contain a more diverse range of linkages, which represent the structure of extracted lignin.

### 1.5 “Hybrid” Model Compounds

The synthesis and use of the type of lignin model compound, which has been discussed thus far in this review, have been extremely beneficial to the scientific community, in terms of adding knowledge and serving as a good starting point for scoping exercises into catalyst refinement, etc. However, there comes a point when we must consider what the actual application of these catalytic systems to raw biomass might look like in terms of reaction conditions, effectiveness of the catalyst and products being formed in the reaction solution. Therefore, the shift must be made to begin to incorporate a new class of model compound which has functionalities shared by the carbohydrate portion of biomass and also of lignin. Initially, the identification of the linkages which are formed between cellulose/hemicellulose and lignin in biomass was an extremely important contribution to the field of biomass conversion.^[^ ^46^ ^]^ This then resulted in the coining of the term, which more accurately describes the functionalities of these crosslinked structures in nature: “Lignin‐Carbohydrate Complex.”^[^ ^47^, ^48^, ^49^, ^50^ ^]^ These elucidation studies within the literature identified a total of six bonding patterns which are found between lignin and cellulose/hemicellulose in nature, with one of the most prevalent being the phenyl glycosidic linkage. There have been very few examples within the literature of these LCC‐type compounds being synthesized as is routinely the case for normal lignin model compounds, even though these models with both carbohydrate and lignin functionalities will give the most realistic view of how raw biomass may act under a given set of catalytic conditions. Our work recently aimed at the recreation of the phenyl glycosidic linkage, resulting in a small library of compounds being synthesized, which shared hemicellulose, cellulose and lignin functionalities.^[^ ^51^ ^]^ This was the first example of this type of linkage being recreated in the literature for the purpose of being a model for catalytic systems; however, there have been examples of ester‐linked model compounds within the literature.^[^ ^52^, ^53^ ^]^ In order for there to be a sustainable method for in‐depth investigative work on lignin conversion, there has to be a shift in the focus toward this new class of lignin models.

As a general conclusion, the synthetic pieces which have been discussed here in this piece of work do represent the breadth of scientific literature with respect to lignin model compounds. Furthermore, the use of relatively simple synthetic chemistry through to the use of sophisticated synthetic techniques (i.e., auxiliaries) shows that the field of model synthesis is a wide‐ranging area of research. Although there have been efforts toward the synthesis of models which have different linkages, the most common present within the literature is indeed the β‐O‐4. In some cases, the synthetic work has been reported as a standalone report, whereas in other cases, these models have been synthesized and used in subsequent degradation studies. The β‐O‐4 does represent the linkage which is seen most widely in native lignin, and this may be a reason for its prolific reporting within the literature, as well as the fact that, in most cases, the synthetic methods are relatively straightforward and follow a general protocol. Moving forward with lignin model synthesis, it is still a key area of research, and as such, the push toward more representative structures with a diverse range of linkages, which even more closely resemble lignin and even biomass as a whole, may be the next step. Therefore, the move toward these new model compounds, which share functionalities found in both lignin and carbohydrate portions of biomass, will be the future of model chemistry because it will give the most realistic view of how raw biomass may act under a given catalytic system.

### 1.6 Lignin Model Compound Degradation Studies

Numerous methods throughout the literature have been employed for the depolymerization and degradation of lignin model compounds.^[^ ^54^ ^]^ The next few sections will provide an overview of the work using the different depolymerization systems.

### 1.7 Metal‐Catalyzed Depolymerization

The employment of metal catalysis for the conversion of lignin sources, including poplar lignin and wheat straw, has been explored previously within the literature with varying degrees of success and a wealth of functionalities formed, including quinones and aromatic aldehydes.^[^ ^55^, ^56^, ^57^, ^58^, ^59^ ^]^

Wu et al.^[^ ^60^ ^]^ used ruthenium‐based catalysts for the hydrogenolysis of lignin model compounds, which have the β‐O‐4 functionality, the catalysts tested being [Ru(H)_2_(CO)(PPh_3_)] and [Ru(H)_2_(CO)(Xantphos)]. The first assertion by the group was that the Xantphos‐based catalyst was the more catalytically active of the two, producing the greatest yields for the hydrogenolysis reactions from a greater range of substrates.

**Scheme 9:**
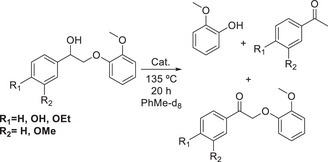
Ruthenium complex catalyzed hydrogenolysis of a simple β‐O‐4 lignin model compound to provide three products overall.^[^ ^60^ ^]^

Some other key work involving Cobalt nanoparticle‐catalyzed hydrogenolysis of β‐O‐4 model compounds was reported by Wu et al.^[^ ^61^ ^]^ In this report, the group demonstrated that the use of this catalytic system was selective for the cleavage of the β‐C‐O ether linkage and also a Clemmensen‐reduction‐type transformation, which completely cleaved the carbonyl group at the α‐position with a CH_2_ serving as its replacement. In this study, the different catalysts, which were tested, included iron, copper, nickel, and cobalt nanoparticles, each of which provided a conversion of substrate greater than 99.2%. Although there have been numerous reports of reactions of this type utilizing lignin model compounds,^[^ ^62^, ^63^, ^64^, ^65^, ^66^, ^67^ ^]^ the field is still growing with new catalysts being reported frequently. Nickel has also been used previously for the depolymerization of lignin and also associated lignin model compounds,^[^ ^68^, ^69^ ^]^ with a notable example having NHC ligands on a nickel center was employed for the cleavage of 4‐O‐5 linkages in lignin under 1 bar pressure of hydrogen, with excellent yields observed.^[^ ^70^ ^]^ The employment of a rare earth metal oxide has also been previously reported by Dong et al., where europium oxide was able to cleave numerous bonds in a dimeric lignin model compound after initially facilitating the oxidation of the benzylic alcohol functionality.^[^ ^71^ ^]^

**Scheme 10:**
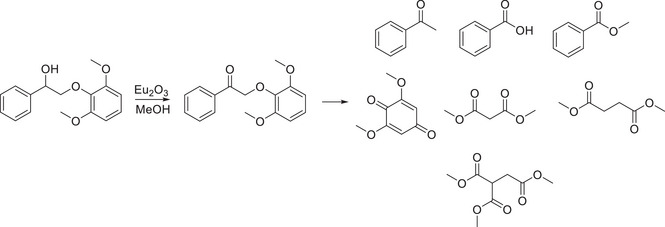
Use of europium oxide for various cleavages and oxidations within the dimer lignin model compound.^[^ ^71^ ^]^

### 1.8 Photocatalytic Valorization

The direct photocatalytic conversion of raw and native lignin has itself been explored^[^ ^72^ ^]^ within the literature with varying results and yields of products including catechol, vanillin, and volatile acids.^[^ ^73^, ^74^, ^75^, ^76^ ^]^ However, one of the main factors which will always hinder the direct photocatalysis of lignin is the lack of real solubility of the substrate and as a direct result, the turbidity of the system. These two issues in tandem make the photocatalytic valorization of lignin a real issue when any form of scale is considered.

The photocatalysis of lignin models is an area which has expanded significantly in the past 20 years, with significant advances being made in terms of substrate scope, complexity of catalytic systems and selectivities.^[^ ^77^ ^]^ A Web Of Science search of the terms “photocatalysis” and “lignin model” returned 90 search results, the majority of which were in the late 2010s. In terms of the types of model compounds which have been investigated in the literature, there is some diversity when it comes to the linkages which have been investigated; however, as expected, the majority of those studies involve β‐O‐4 models. Within the literature, there are only two examples of a 5‐5′ model compound being investigated employing TiO_2_ as the catalytic system.^[^ ^73^, ^78^ ^]^ It was observed in both cases that there was only reaction on the aromatic rings or the sidechains and no cleavage of the central C─C linkage was observed.

**Scheme 11:**
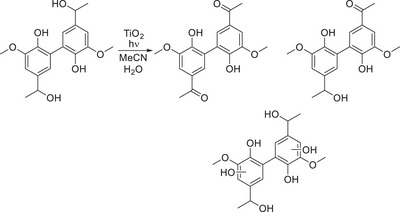
Titanium dioxide mediated photocatalytic degradation of a 5‐5′ lignin model compound by Murnaghan et al.^[^ ^78^ ^]^

Within this study by Murnaghan et al. the same catalytic system was extended to a lignin model hexamer, and it was observed by the group that the complete consumption of the oligomeric model occurred within ≈90 min and a total of nine products being formed and elucidated through the use of LC‐MS.

**Scheme 12:**
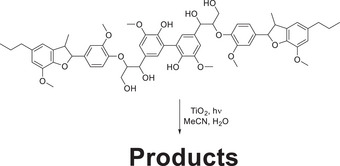
Hexameric lignin model compound employed in study by Murnaghan et al.^[^ ^78^ ^]^

Within the same group, an investigation into the β‐5 linkage in a model dimer was explored by Murnaghan in another study.^[^ ^79^ ^]^ It was observed by the group that within the first 2 min of irradiation of the solution, there was the formation of a diol species in the alkene sidechain. Moving toward the more common β‐O‐4 model compounds, there have been significant advancements in the use of photocatalytic strategies for cleavage within models of this type.^[^ ^80^, ^81^, ^82^, ^83^, ^84^ ^]^ For this photocatalytic work, which was done in an acetonitrile/water mixture, there is undoubtedly a role that the organic solvent is playing in the mechanism and contributing in some capacity to the conversion process of the lignin model. In our work focused on the β‐5 dimer, we devised a mechanistic proposal which contributes to the understanding of how acetonitrile behaves under the photocatalytic conditions.

Crestini et al. reported the use of the singlet oxygen sensitizer Rose Bengal for the degradation of a β‐O‐4 model compound.^[^ ^85^, ^86^ ^]^ This type of work was also studied by Barclay et al. where the effect of the singlet oxygen on numerous lignin models investigated the yellowing of paper.^[^ ^87^ ^]^

**Scheme 13:**
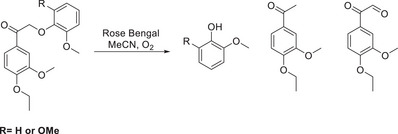
Employment of Singlet Oxygen sensitizer Rose Bengal for the degradation of β‐O‐4 model compound.^[^ ^85^, ^86^ ^]^

Interestingly, Crestini et al. proposed that the mechanism for this conversion goes through an *endo*peroxide species where the aromaticity of the B‐ring in the model compound is broken, and this begins a cleavage process between the β─C─O bond and the aromatic ring.

Using a graphitic carbon nitride photocatalyst, Liu et al.^[^ ^88^ ^]^ investigated the cleavage of C─C bonds within β‐O‐4 and β‐1 model compounds. The group noted that the formation of π‐π stacking interactions between the aromatic substrate and the NH_x_ groups, which have formed on the surface of the catalyst, where photogenerated holes were the dominant active species, thereafter, in the transformation of the substrates. Graphitic carbon nitride has also been employed in our own studies for the conversion of a β‐5 lignin model dimer, with the products proposed to be vanillin and a range of other benzofuran entities.^[^ ^89^ ^]^ Some other notable pieces of work involving the use of photocatalytic strategies for the conversion of lignin models are shown in these refs. [90, 91, 92]

An interesting piece of work by Xu et al demonstrated that the use of triphenylpyrilium tetrafluoroborate and CaCl_2_ could be used under blue LED irradiation to generate a chlorine radical, which goes on to cleave the α─β C─C bond to generate carboxylic acid and phenolic formate esters.^[^ ^93^ ^]^

The employment of a similar dimer containing a β‐5 type linkage was employed for photocatalytic reaction with graphitic^[^ ^94^ ^]^ carbon nitride, where it was observed that the formation of vanillin and cyanoguaiacol were among the major components of the reaction solution following irradiation. This is an interesting finding in the form of the cyano derivative, as the reaction, which has been run in acetonitrile there has been a reaction between the photocatalytic conditions and the solvent, and this reactive intermediate has, in some capacity, reacted with the intermediates in the reaction solution.

**Figure 7:**
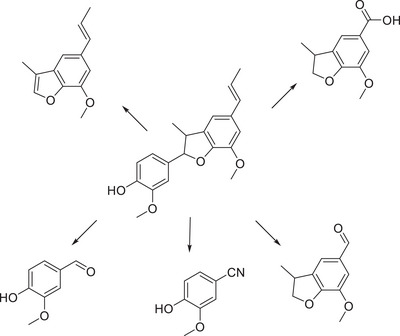
*G*‐C_3_N_4_ photocatalyzed the conversion of the dimer with a β‐5 linkage toward numerous products.^[^ ^94^ ^]^

The future of lignin model photocatalysis appears to be one which will remain fruitful, especially through the use of photoredox^[^ ^83^, ^92^, ^95^ ^]^ and decorated photocatalysts,^[^ ^94^, ^97^ ^]^ which may aid in the increase of the bandgap and prevent recombination of the electron with the formed hole in the valence band.

### 1.9 Transition Metal and Metal Porphyrin Catalysis

There have been numerous reports of the use of vanadium complexes in the presence of oxygen for the cleavage of linkages within lignin model compounds.^[^ ^98^, ^99^, ^100^ ^]^ In the references provided here, the mixture of products which were received as a result of the oxidative cleavages also provided products which have a benzyl carbonyl group. This finding is in good agreement with the assertion by Beckham et al.^[^ ^101^ ^]^ whereby in the case of a β‐O‐4 linkage, the oxidation of the benzylic alcohol will result in the weakening of the β─C─O bond. Copper‐catalyzed oxidation of β‐O‐4 model compounds bearing diol functionalities has been shown to also proceed through this benzylic oxidation pathway.^[^ ^102^ ^]^ Smaller model compounds (veratryl alcohol and 2,2′biphenol) were exposed to a copper complex in the presence of O_2_ (8 bar) in a study by Sippola et al.^[^ ^103^ ^]^ It was claimed that during the process, the generation of hydrogen peroxide is the preceding event for the mechanisms of oxidation. The use of hydrogen peroxide as the terminal oxidant with a transition metal catalyst has also been explored by Crestini et al.^[^ ^104^ ^]^ for the degradation of veratryl alcohol and a dimeric lignin model compound with a β‐O‐4 linkage.

It was observed that during these degradation reactions, there was significant oxidation of the substrate and the subsequent intermediates formed, resulting in the formation of quinones and muconolactone species. Noteworthy during this study was the fact that when a nonphenolic β‐O‐4 dimer was subjected to the catalytic system, there was no formation of the muconolactone species. Furthermore, the system was extended to a diphenylmethane model dimer with cleavage of the central linkage to provide a carboxylic acid functionality.

**Scheme 14:**
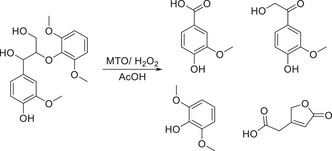
Employment of MTO/H_2_O_2_ catalytic system for the conversion of dimeric β‐O‐4 lignin model compound toward oxidation products.^[^ ^104^ ^]^

Biomimetic approaches toward lignin model degradation have been explored by numerous researchers, with a range of different porphyrin catalysts being synthesized and employed for the conversion reactions of different model compounds.^[^ ^59^, ^105^, ^106^, ^107^ ^]^ Studies by Crestini^[^ ^107^ ^]^ on monomers and dimers in the presence of iron and manganese porphyrins with varying R groups on the porphyrin ring have been reported. Again, the comparison of phenolic and nonphenolic β‐O‐4 model dimers with a difference in the products being afforded, other substrates investigated were the 5‐5′ biphenyl, diphenylmethane, with varying levels of oxidation of products and yields being provided from the catalytic system. The investigation of the β‐5 model dimer is an area of literature which is relatively sparse; however, some significant work by Dolphin et al.^[^ ^106^ ^]^ showed that the employment of an iron porphyrin for the oxidation of lignin model compounds, including the β‐5 linked dimer.

**Figure 8:**
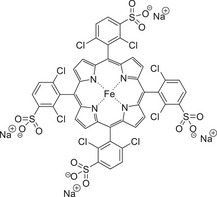
Structure of iron porphyrin employed by Dolphin^[^ ^106^ ^]^ for the oxidation of lignin model compounds.

Focusing on the β‐5 linked dimeric model, a nonphenolic moiety was studied, and it was seen that there were numerous oxidation and cleavage events occurring within the model compound. In terms of the reactivity of the compound, it can be seen that the sites of enhanced reactivity are the alkene in the propene sidechain and also the benzofuran ring at the center of the compound.

**Scheme 15:**
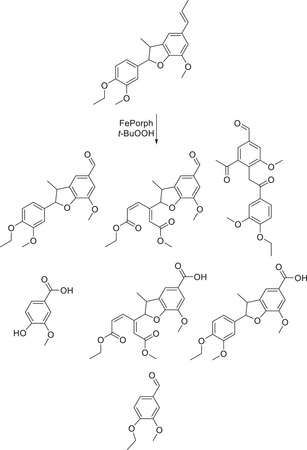
Range of products provided by β‐5 dimer.^[^ ^106^, ^108^ ^]^

### 1.10 Ionic Liquid Mediated Valorization

**Figure 9:**
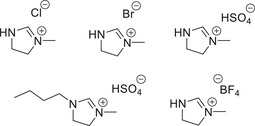
Range of acidic ionic liquids employed in the study by Cox et al.^[^ ^117^ ^]^

Ionic liquids have the unique ability to solvate and facilitate certain reactions under a provided set of conditions. These tunable liquids have been extended to lignin^[^ ^109^, ^110^, ^111^ ^]^ and lignin model compounds^[^ ^112^, ^113^, ^114^, ^115^ ^]^ for certain cleavages within the compounds. Almost all of the examples of ionic liquids being employed for the conversion of lignin model compounds have studied the β‐O‐4 model compound. The oxidative cleavage of a model dimer employing O_2_ as a terminal oxidant was studied by Yang et al.,^[^ ^116^ ^]^ where the ionic liquid [OMIM][OAc] was compared to toluene as the reaction solvent and a disparity of 84% conversion was observed between the two solvents the IL being the better. Comparison of this work with a study by Cox et al.^[^ ^117^ ^]^ where two model dimers were studied in a range of acidic ionic liquids (Figure 9). The employment of these acidic ionic liquids facilitated the degradation of the model dimers, and pathways for conversion via hydrolysis of the ether linkage were proposed by the group. Employing ionic liquids as a reaction medium for the implementation of an electrochemical process has also been explored for the cleavage of linkages within lignin model compounds.^[^ ^118^ ^]^

### 1.11 Enzyme‐Mediated Valorization

The biosynthesis of lignin precursors in nature is a process which is completely governed by enzymes, and the initiation of the biosynthesis of lignin is also dominated by the presence of enzymes. The use, therefore, of enzymes for the degradation of lignin^[^ ^119^, ^120^, ^121^, ^122^, ^123^, ^124^, ^125^, ^126^ ^]^ and lignin models has been reported extensively also. Most notable is the use of three different enzymes:‐ Lignin peroxidase (LiP),^[^ ^127^, ^128^ ^]^ Manganese peroxidase (MnP)^[^ ^129^, ^130^, ^131^ ^]^ and Laccase (LA),^[^ ^132^, ^133^, ^134^ ^]^ but other enzymes have also been reported.^[^ ^135^ ^]^ One of the key characteristics of lignin as a whole is the presence of methoxy groups (OMe) bonded directly to aromatic rings. Some work by Lopretti et al.^[^ ^136^ ^]^ showed that enzyme extracts taken from the fungus *Gloeophilum trabeum* were successful in the demethoxylation reaction of lignin model compounds veratraldehyde, veratryl alcohol, and anisoin. Using analytical techniques, the formation of phenolic products was determined owing to the formation of methanol in the reaction solution as detected by HPLC. Another study by Ohta et al.^[^ ^137^ ^]^ demonstrated that the use of six enzymes (short‐chain dehydrogenases) from a strain of Novosphingobium has the ability to convert stereoisomeric β‐O‐4 model compounds to monomeric units. The pathway, the group, and report begin with the oxidation of the benzylic alcohol and subsequent cleavage to result in the monomeric units guaiacol and guaiacylhydroxylpropanone.

**Scheme 16:**
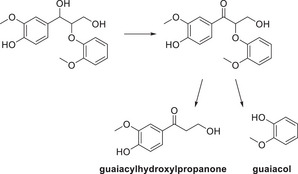
Conversion of a β‐O‐4 model compound by the combination of six enzymes isolated from marine Novosphingobium.^[^ ^137^ ^]^

Laccases, as previously mentioned, have been instrumental in research focusing on the enzymatic degradation of lignin model compounds. It has been reported that the use of laccase with a monomeric lignin model compound results in the cleavage of the aromatic ring and the formation of a muconolactone species.^[^ ^138^ ^]^ The group proposes that there are two possible pathways for the formation of the lactone species, with both pathways beginning with the phenoxyl radical formation and subsequent reaction with molecular oxygen.

Along with the use of a mediator, which results in a slightly different mechanism of degradation, laccase was employed for the oxidation of veratryl alcohol to veratraldehyde.^[^ ^139^ ^]^ During this study, veratryl alcohol and laccase were mixed for 18 h, and the total amount of veratraldehyde produced was 0.04 mm. However, with a mediator present with the enzyme and substrate, the concentration of veratraldehyde after 18 h was 0.99 mm. The relative lack of reactivity of the laccase toward the veratryl alcohol is due to the substrate bearing no phenolic functionalities, which laccase would normally react with. Alongside the previous example of laccase being employed for the conversion of lignin model compounds, our own lab showed how a laccase/1‐HBT system could efficiently cleave linkages in the β‐O‐4 sidechain of a hexameric lignin model compound to provide a range of products and complete consumption of the hexamer substrate within 24 h.^[^ ^140^ ^]^

Mentioned previously was the use of manganese peroxidase in the conversion of lignin model compounds, when reacted with two β‐O‐4 model compounds (with and without a benzylic carbonyl), it was observed that a number of products were formed.^[^ ^129^ ^]^ The model bearing an alcohol at the benzylic position was oxidized at the benzylic position, with the yield being 50%. Following this, there were other cleavage events to provide lower‐yielding monomeric units.

**Scheme 17:**
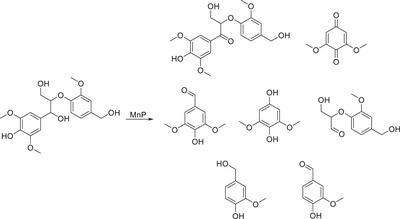
Employment of manganese peroxidase for the conversion of a phenolic β‐O‐4 dimeric model toward a plethora of products.^[^ ^129^ ^]^

Moving beyond LiP, MnP, and laccase, the utilization of the immobilized lipase Novozyme 435 for the cleavage of C─C bonds in a ketonic lignin model compound and most importantly, it has been reported by Zhang et al. that the Novozyme 435 biocatalyst shows good recyclability in the process.^[^ ^141^ ^]^

## 2 Conclusion

The current state of the literature with regard to the synthesis of lignin model compounds is one which is significantly broad in terms of work which has been completed; however, there are narrow avenues when the breadth of work within the synthetic ventures is considered, with the bulk of the work focusing on β‐O‐4 linkages. This is a shortcoming of the literature of this field, but advances have been made in terms of moving forward with complexity and the representative nature of model compounds. Especially when the synthetic routes toward lignin‐carbohydrate complex model compounds are considered, the sheer complexity of these linkages leaves open a new avenue for the community to explore fully and develop the best models which can be employed for catalytic studies. This, we believe, is where the real future of model compound chemistry lies, and as such, there has to be significant focus given to this class of model with the next steps being the development of catalytic systems for their conversion. Moving from synthesis to conversion and depolymerization, the vast majority of the literature relies on the cleavage of ether bonds within lignin models. Undoubtedly, this is due to those being the weakest in a model compound and, as such, will have a higher likelihood of being the first one to go. However, even our own work in the photocatalytic conversion of the lignin model hexamer highlighted a shortcoming of the photocatalytic technology–it was inefficient at the cleavage of C─C bonds, especially the 5‐5′ linkage, which has the highest enthalpy of all bonds present in lignin. If there is to be a significant leap forward in the understanding and development of lignin conversion, more attention must be paid to the models bearing a 5‐5′ linkage. Conversely, if we as a community can get to a point where the catalytic systems being deployed for biomass conversion will cleave all bonds but leave behind 5‐5′ biphenyl linkages, we have indirect selectivity as a result of incapability, which is itself a development.

On a more holistic note, the role which is played by biomass is massively uncertain purely because we have no direct means for its conversion, the chest of gold but no key is the real issue when it comes to sustainability. The development of technology which can unlock the nature of lignin and depolymerize so that monomeric units can be produced with ease is the ultimate goal, and we are still some time off from having a robust protocol for this means. However, one thing that we can say with certainty is that lignin model chemistry will be here for many years to come in this regard.

## Conflict of Interests

The authors declare no conflict of interest.

## Data Availability

Data sharing is not applicable to this article as no new data were created or analyzed in this study.

## References

[1] A. V. Balatsky , G. I. Balatsky , S. S. Borysov , Sustainability (Switzerland) 2015, 7, 3430.

[2] R. G. Miller , S. R. Sorrell , H. Lane , S. Kt , R. G. Miller , Phil. Trans. R. Soc. A. 2013, 372, 20130179.24298085

[3] R. James , Curr. Anthropol. 1989, 30, 1.

[4] R. Makino , S. Ohara , K. Hashida , J. Tropical Forest Sci. 2009, 21, 45.

[5] A. E. Grazhdannikov , L. M. Kornaukhova , V. I. Rodionov , N. A. Pankrushina , E. E. Shults , A. S. Fabiano‐Tixier , S. A. Popov , F. Chemat , ACS Sustainable Chem. Eng. 2018, 6, 6281.

[6] I. Mota , P. C. Rodrigues Pinto , C. Novo , G. Sousa , O. Guerreiro , Â. R. Guerra , M. F. Duarte , A. E. Rodrigues , Ind. Eng. Chem. Res. 2012, 51, 6991.

[7] T. Werpy , G. Petersen , in Top Value Added Chemicals from Biomass Volume, 2004, Pacific Northwest National Laboratory.

[8] G. G. Gross , Adv. Bot. Res. 1981, 8, 25.

[9] C. Canevali , M. Orlandi , L. Pardi , B. Rindone , R. Scotti , J. Sipila , F. Morazzoni , J. Chem. Soc., Dalton Trans. 2002, 0, 3007.

[10] E. Novaes , M. Kirst , V. Chiang , H. Winter‐Sederoff , R. Sederoff , Plant Physiol. 2010, 154, 555. 20921184 10.1104/pp.110.161281 PMC2949025

[11] J. Zakzeski , P. C. A. Bruijnincx , A. L. Jongerius , B. M. Weckhuysen , Chem. Rev. 2010, 110, 3552. 20218547 10.1021/cr900354u

[12] A. K. Sangha , J. M. Parks , R. F. Standaert , A. Ziebell , M. Davis , J. C. Smith , J. Phys. Chem. B 2012, 116, 4760. 22475051 10.1021/jp2122449

[13] S. Guadix‐Montero , M. Sankar , Top. Catal. 2018, 61, 183.

[14] H. Kawamoto , J. Wood Sci. 2017, 63, 117.

[15] C. S. Lancefield , N. J. Westwood , Green Chem. 2015, 17, 4980.

[16] F. Tran , C. S. Lancefield , P. C. J. Kamer , T. Lebl , N. J. Westwood , Green Chem. 2015, 17, 244.

[17] J. C. Jung , O. S. Park , Synth. Commun. 2007, 37, 1665.

[18] W. G. Forsythe , M. D. Garrett , C. Hardacre , M. Nieuwenhuyzen , G. N. Sheldrake , Green Chem. 2013, 15, 3031.

[19] Z. Fang , M. S. Meier , Org. Biomol. Chem. 2018, 16, 2330. 29542792 10.1039/c8ob00409a

[20] S. Li , K. Lundquist , Acta Chem. Scand. 1997, 51, 1224.

[21] E. Adler , E. Eriksoo , Acta Chem. Scand. 1955, 9, 341.

[22] B. O. L. U. S. E. Adler , Svensk Pepperstidn 1952, 55, 245.

[23] S. R. K. Lundquist , Acta Chem. Scand. 1975, B29, 276.

[24] K. K. H. K. J. N. S. Hosoya , Mokuzai Gakkaishi 1980, 26, 118.

[25] F. N. T. H. T. Katayama , Mozukai Gakkaishi 1981, 27, 223.

[26] G. E. Miksche , Acta Chem. Scand. 1966, 20, 1038.

[27] A. Mukhtar , M. Zaheer , M. Saeed , W. Voelter , Zeitschrift fur Naturforschung – Sec. B J. Chem. Sci. 2017, 72, 119.

[28] T. Kishimoto , Y. Uraki , M. Ubukata , Synthesis (Stuttg) 2005, 1067. 10.1039/b416699j 15750650

[29] J. Buendia , J. Mottweiler , C. Bolm , Chem. – A Eur. J. 2011, 17, 13877. 10.1002/chem.201101579 22076640

[30] C. N. Njiojob , J. J. Bozell , B. K. Long , T. Elder , R. E. Key , W. T. Hartwig , Chem. – A Eur. J. 2016, 22, 12506. 10.1002/chem.201601592 27459234

[31] S. Hishiyama , Y. Otsuka , M. Nakamura , S. Ohara , S. Kajita , E. Masai , Y. Katayama , Tetrahedron Lett. 2012, 53, 842.

[32] K. Li , R. F. Helm , Holzforschung 2000, 54, 597.

[33] U. Weißbach , S. Dabral , L. Konnert , C. Bolm , J. G. Hernández , Beilstein J. Org. Chem. 2017, 13, 1788. 28904622 10.3762/bjoc.13.173 PMC5588541

[34] P. Karhunen , P. Rummakko , A. Pajunen , G. Brunow , J. Chem. Soc. Perkin 1 1996, 4, 2303.

[35] P. Karhunen , P. Rummakko , J. Sipilä , G. Brunow , I. Kilpeläinen , Tetrahedron Lett. 1995, 36, 4501.

[36] B. Borecka , T. S. Cameron , A. Linden , P. R. Ranjbar , J. Sandstrdm , J. Am. Chem. Soc. 1990, 112, 1185.

[37] C. Canevali , F. Morazzoni , M. Orlandi , B. Rindone , R. Scotti , J. Sipila , G. Brunow , Oxidative Delignification Chemistry 2001, 785, 197.

[38] M. Lahtinen , A. Haikarainen , J. Sipilä , Holzforschung 2013, 67, 129.

[39] S. Ciofi‐Baffoni , L. Banci , A. Brandi , J. Chem. Soc.‐Perkin Trans. 1 1998, 2, 3207.

[40] C. W. Lahive , P. J. Deuss , C. S. Lancefield , Z. Sun , D. B. Cordes , C. M. Young , F. Tran , A. M. Z. Slawin , J. G. De Vries , P. C. J. Kamer , N. J. Westwood , K. Barta , J. Am. Chem. Soc. 2016, 138, 8900. 27310182 10.1021/jacs.6b04144

[41] F. Yue , F. Lu , S. Ralph , J. Ralph , Biomacromolecules 2016, 17, 1909. 27078826 10.1021/acs.biomac.6b00256

[42] R. Katahira , H. Kamitakahara , T. Takano , F. Nakatsubo , Journal of Wood Science 2006, 52, 255.

[43] T. Kishimoto , Y. Uraki , M. Ubukata , Org. Biomol. Chem 2006, 4, 1343. 16557323 10.1039/b518005h

[44] T. Kishimoto , Y. Uraki , M. Ubukata , J. Wood Chem. Technol. 2008, 28, 97.

[45] T. Kishimoto , Y. Uraki , M. Ubukata , Org. Biomol. Chem. 2008, 6, 2982. 18688492 10.1039/b805460f

[46] Y. Miyagawa , T. Mizukami , H. Kamitakahara , T. Takano , Holzforschung 2014, 68, 747.

[47] D. Tarasov , M. Leitch , P. Fatehi , Biotechnol. Biofuels 2018, 11, 1. 30288174 10.1186/s13068-018-1262-1 PMC6162904

[48] A. Björkman , Ind. Eng. Chem. 1957, 49, 1395.

[49] Y. Xie , S. Yasuda , H. Wu , H. Liu , J. Wood Sci. 2000, 46, 130.

[50] M. Balakshin , E. Capanema , A. Berlin , in Isolation and analysis of lignin‐carbohydrate complexes preparations with traditional and advanced methods: A Review 2014, Elsevier B.V., 1st ed., 42.

[51] G. N. Sheldrake , N. M. Curran , C. W. J. Murnaghan , Biomass and Bioenergy 2025, 193, 107555.

[52] K. Li , R. F. Helm , J. Agric. Food Chem. 1995, 43, 2098.

[53] M. Toikka , J. Sipilä , A. Teleman , G. Brunow , J. Chem. Soc. Perkin 1998, 1, 3813.

[54] C. W. Lahive , P. C. J. Kamer , C. S. Lancefield , P. J. Deuss , Chemsuschem 2020, 13, 4238. 32510817 10.1002/cssc.202000989 PMC7540175

[55] J. M. W. Chan , S. Bauer , H. Sorek , S. Sreekumar , K. Wang , F. D. Toste , ACS Catal. 2013, 3, 1369.

[56] B. Biannic , J. J. Bozell , Org. Lett. 2013, 15, 2730. 23679189 10.1021/ol401065r

[57] R. Sun , J. M. Lawther , W. B. Banks , Ind. Crops Prod. 1995, 4, 241.

[58] M. P. Masingale , E. F. Alves , T. N. Korbieh , S. K. Bose , R. C. Francis , BioResources 2009, 4, 1139.

[59] J. Zeng , G. Yoo , F. Wang , X. Pan , W. Vermerris , T. Zhaohui , Chemsuschem 2015, 8, 861. 25663189 10.1002/cssc.201403128

[60] A. Wu , B. O. Patrick , E. Chung , B. R. James , Dalton Trans. 2012, 41, 11093. 22864631 10.1039/c2dt31065a

[61] W. Wu , H. Liu , H. Wu , B. Zheng , S. Han , K. Zhang , X. Mei , C. Xu , M. He , B. Han , ACS Sustainable Chem. Eng. 2021, 9, 11862.

[62] H. T. Wang , Z. K. Li , H. L. Yan , Z. P. Lei , J. C. Yan , S. B. Ren , Z. C. Wang , S. G. Kang , H. F. Shui , Fuel 2022, 326, 125027.

[63] L. Jiang , H. Guo , C. Li , P. Zhou , Z. Zhang , Chem. Sci. 2019, 10, 4458. 31057773 10.1039/c9sc00691e PMC6482439

[64] J. He , C. Zhao , J. A. Lercher , J. Am. Chem. Soc. 2012, 134, 20768. 23190332 10.1021/ja309915e

[65] V. Molinari , C. Giordano , M. Antonietti , D. Esposito , J. Am. Chem. Soc. 2014, 136, 1758. 24437507 10.1021/ja4119412

[66] J. F. H. F. Gao , J. D. Webb , Angew. Chem. – Int. Ed. 2016, 55, 1474. 10.1002/anie.201509133 26666391

[67] J. Liang , M. X. Wang , Y. P. Zhao , W. W. Yan , X. G. Si , G. Yu , J. P. Cao , X. Y. Wei , Chemcatchem 2021, 13, 3836.

[68] W. Schutyser , S. Van den Bosch , J. Dijkmans , S. Turner , M. Meledina , G. Van Tendeloo , D. P. Debecker , B. F. Sels , ChemSusChem 2015, 8, 1805. 25881563 10.1002/cssc.201403375

[69] H. Zhao , C. Cheng , B. Zhu , Y. Yang , Q. Wang , D. Shen , X. Jiang , Biomass and Bioenergy, 2024, 184, 107186.

[70] A. G. Sergeev , J. F. Hartwig , Science 1979, 2011, 332, 439. 10.1126/science.1200437 21512027

[71] Q. Dong , Z. Tian , W. Song , W. Deng , H. Zhang , Colloids Surf A Physicochem Eng Asp, 2021, 626, 126846.

[72] P. J. Deuss , K. Barta , Elsevier, 2016, Coordination Chemistry Reviews, 306, 510.

[73] R. Prado , X. Erdocia , J. Labidi , Chemosphere 2013, 91, 1355. 23473431 10.1016/j.chemosphere.2013.02.008

[74] K. Tanaka , R. C. R. Calanag , T. Hisanaga , J. Mol. Catal. A Chem. 1999, 138, 287.

[75] Y. S. Ma , C. N. Chang , Y. P. Chiang , H. F. Sung , A. C. Chao , Chemosphere 2008, 71, 998. 18093635 10.1016/j.chemosphere.2007.10.061

[76] S. K. Kansal , M. Singh , D. Sud , J. Hazard. Mater. 2008, 153, 412. 17936502 10.1016/j.jhazmat.2007.08.091

[77] N. Skillen , H. Daly , L. Lan , M. Aljohani , C. W. J. Murnaghan , X. Fan , C. Hardacre , G. N. Sheldrake , P. K. J. Robertson , 2022, Topics in Current Chemistry, 380, 1. 10.1007/s41061-022-00391-9 PMC9206627 35717466

[78] C. W. J. Murnaghan , N. Skillen , B. Hackett , J. Lafferty , P. K. J. Robertson , G. N. Sheldrake , ACS Sustainable Chem. Eng. 2022, 10, 12107. 36161097 10.1021/acssuschemeng.2c01606 PMC9490757

[79] C. W. J. Murnaghan , N. Skillen , C. Hardacre , J. Bruce , G. N. Sheldrake , P. K. J. Robertson , J. Phys.: Energy 2021, 3, 1.

[80] M. D. Kärkäs , B. S. Matsuura , T. M. Monos , G. Magallanes , C. R. J. Stephenson , Org. Biomol. Chem. 2016, 14, 1853. 26732312 10.1039/c5ob02212f

[81] J. D. Nguyen , B. S. Matsuura , C. R. J. Stephenson , J. Am. Chem. Soc. 2014, 136, 1218. 24367945 10.1021/ja4113462

[82] N. Luo , M. Wang , H. Li , J. Zhang , H. Liu , F. Wang , ACS Catal. 2016, 6, 7716.

[83] J. Zhang , Chemsuschem 2018, 11, 3071. 29989337 10.1002/cssc.201801370

[84] Z. Xiang , W. Han , J. Deng , W. Zhu , Y. Zhang , H. Wang , Chemsuschem 2020, 13, 4199. 32329562 10.1002/cssc.202000601

[85] C. Crestini , M. D'Auria , J. Photochem. Photobiol. A Chem. 1996, 101, 69.

[86] C. Crestini , M. D'Auria , Tetrahedron 1997, 53, 7877.

[87] M. Barclay , L. R. C. Grandy , J. K. MacKinnon , H. D. Nichol , H. C. Vinqvist , Can. J. Chem. 1998, 76, 1805.

[88] H. Liu , H. Li , J. Lu , S. Zeng , M. Wang , N. Luo , S. Xu , F. Wang , ACS Catal. 2018, 8, 4761.

[89] J. Liu , K. Ralphs , C. Murnaghan , N. Skillen , G. Sheldrake , P. McCarron , P. K. J. Robertson , Chemsuschem, 2025, 18, e202400955. 39255046 10.1002/cssc.202400955 PMC11789975

[90] W. Li , M. Zhang , Z. Du , H. Jameel , H. Chang , BioResources 2015, 10, 1245.

[91] J. Chen , W. Liu , Z. Song , H. Wang , Y. Xie , BioEnergy Res. 2018, 11, 166.

[92] J. Luo , X. Zhang , J. Lu , J. Zhang , ACS Catal. 2017, 7, 5062.

[93] E. Xu , F. Xie , T. Liu , J. He , Y. Zhang , Chem. – A Eur. J., 2024, 30, e202304209. 10.1002/chem.202304209 38372165

[94] J. Liu , K. Ralphs , C. Murnaghan , N. Skillen , G. Sheldrake , P. McCarron , P. K. J. Robertson , Chemsuschem 2024, 18, 1. 10.1002/cssc.202400955 PMC11789975 39255046

[95] M. D. Kärkäs , I. Bosque , B. S. Matsuura , C. R. J. Stephenson , Org. Lett. 2016, 18, 5166. 27662412 10.1021/acs.orglett.6b02651

[96] X. Liu , X. Duan , W. Wei , S. Wang , B. J. Ni , Green Chem. 2019, 21, 4266.

[97] S. Li , S. Liu , J. C. Colmenares , Y. Xu , Green Chem. 2016, 18, 594.

[98] B. Sedai , C. Diaz‐Urrutia , R. T. Baker , R. Wu , L. A. P. Silks , S. K. Hanson , ACS Catal. 2013, 3, 3111.

[99] S. K. Hanson , R. T. Baker , J. C. Gordon , B. L. Scott , D. L. Thorn , Inorg. Chem. 2010, 49, 5611. 20491453 10.1021/ic100528n

[100] B. Sedai , C. Díaz‐Urrutia , R. T. Baker , R. Wu , L. A. P. Silks , S. K. Hanson , ACS Catal. 2011, 1, 794.

[101] S. Kim , S. C. Chmely , M. R. Nimlos , Y. J. Bomble , T. D. Foust , R. S. Paton , G. T. Beckham , J. Phys. Chem. Lett. 2011, 2, 2846.

[102] H. E. P. Salonen , C. P. A. Mecke , M. I. Karjomaa , P. M. Joensuu , A. M. P. Koskinen , ChemistrySelect 2018, 3, 12446.

[103] V. O. Sippola , A. O. I. Krause , Catal. Today 2005, 100, 237.

[104] C. Crestini , M. C. Caponi , D. S. Argyropoulos , R. Saladino , Bioorg. Med. Chem. 2006, 14, 5292. 16621577 10.1016/j.bmc.2006.03.046

[105] X. F. Zhou , X. J. Lu , J. Appl. Polym. Sci., 2016, 133, 1.

[106] F. Cui , D. Dolphin , Bioorg. Med. Chem 1994, 2, 735. 7858983 10.1016/0968-0896(94)85025-9

[107] C. Crestini , A. Pastorini , P. Tagliatesta , J. Mol. Catal. A Chem. 2004, 208, 195.

[108] F. Cui , D. Dolphin , Can. J. Chem. 1995, 73, 2153.

[109] A. Diop , K. Jradi , C. Daneault , D. Montplaisir , BioResources 2015, 10, 4933.

[110] A. Diop , A. H. Bouazza , C. Daneault , D. Montplaisir , BioResources 2013, 8, 4270.

[111] K. Stärk , N. Taccardi , A. Bösmann , P. Wasserscheid , Chemsuschem 2010, 3, 719. 20480494 10.1002/cssc.200900242

[112] J. B. Binder , M. J. Gray , J. F. White , Z. C. Zhang , J. E. Holladay , Biomass and Bioenergy 2009, 33, 1122.

[113] W. E. S. Hart , L. Aldous , J. B. Harper , ChemPlusChem 2018, 83, 348. 31957355 10.1002/cplu.201700486

[114] S. G. Yao , M. S. Meier , R. B. Pace , M. Crocker , RSC Advances 2016, 6, 104742.

[115] Y. Yang , H. Fan , J. Song , Q. Meng , H. Zhou , L. Wu , G. Yang , B. Han , Chem. Commun. 2015, 51, 4028. 10.1039/c4cc10394g 25661479

[116] Y. Yang , H. Fan , Q. Meng , Z. Zhang , G. Yang , B. Han , Chem. Commun. 2017, 53, 8850. 10.1039/c7cc04209d 28737186

[117] S. Jia , B. J. Cox , X. Guo , Z. C. Zhang , J. G. Ekerdt , Ind. Eng. Chem. Res. 2011, 50, 849.

[118] G. Liu , Q. Wang , D. Yan , Y. Zhang , C. Wang , S. Liang , L. Jiang , H. He , Green Chem. 2021, 23, 1665.

[119] D. S. Arora , M. Chander , P. K. Gill , Int Biodeterior Biodegradation 2002, 50, 115.

[120] K. Johansson , T. Gillgren , S. Winestrand , L. Järnström , L. J. Jönsson , J. Biol Eng 2014, 8, 1. 24382027 10.1186/1754-1611-8-1 PMC3882780

[121] T. D. H. Bugg , T. D. H. Bugg , M. Ahmad , E. M. Hardiman , R. Rahmanpour , Nat. Prod. Rep. 2011, 28, 1883. 21918777 10.1039/c1np00042j

[122] Z. Mycroft , M. Gomis , P. Mines , P. Law , T. D. H. Bugg , Green Chem. 2015, 17, 4974.

[123] M. Ahmad , J. N. Roberts , E. M. Hardiman , R. Singh , L. D. Eltis , T. D. H. Bugg , Biochemistry 2011, 50, 5096. 21534568 10.1021/bi101892z

[124] P. D. Sainsbury , E. M. Hardiman , M. Ahmad , H. Otani , N. Seghezzi , L. D. Eltis , T. D. H. Bugg , ACS Chem. Biol. 2013, 8, 2151. 23898824 10.1021/cb400505a

[125] P. Ander , K.‐E. Eriksson , Arch. Microbiol. 1976, 109, 1.

[126] C. S. Evans , FEMS Microbiol. Lett. 1985, 27, 339.

[127] T. Higuchi , Proc. Jpn. Acad. Ser B Phys. Biol. Sci. 2004, 80, 204.

[128] L. T. Mai Pham , S. J. Kim , Y. H. Kim , Biotechnol. Biofuels 2016, 9, 1. 27872660 10.1186/s13068-016-0664-1 PMC5111271

[129] U. Tuor , H. Wariishi , M. H. Gold , H. E. Schoemaker , Biochemistry 1992, 31, 4986. 1599925 10.1021/bi00136a011

[130] M. Tien , T. K. Kirk , Proc. Natl. Acad. Sci. USA 1984, 81, 2280.16593451

[131] T. K. K. M. Tien , Science 1979, 1983, 221, 661.

[132] T. D. H. Bugg , M. Ahmad , E. M. Hardiman , R. Rahmanpour , Nat. Prod. Rep. 2011, 28, 1883. 21918777 10.1039/c1np00042j

[133] A. M. Barreca , M. Fabbrini , C. Galli , P. Gentili , S. Ljunggren , J. Mol. Catal. B Enzym. 2003, 26, 105.

[134] R. U. Leonowicz , J. M. Edgehill , A. Bollag , Arch. Microbiol. 1984, 137, 89.

[135] E. Rosini , C. Allegretti , R. Melis , L. Cerioli , G. Conti , L. Pollegioni , P. D'Arrigo , Catal. Sci. Technol. 2016, 6, 2195.

[136] M. Lopretti , D. Cabella , J. Morais , A. Rodrigues , Process Biochem. 1998, 33, 657.

[137] Y. Ohta , S. Nishi , R. Hasegawa , Y. Hatada , Sci. Rep. 2015, 5, 2. 10.1038/srep15105 PMC4609964 26477321

[138] S. Kawai , T. Umezawa , M. Shimada , T. Higuchi , FEBS Lett. 1988, 236, 309. 3410044 10.1016/0014-5793(88)80043-7

[139] R. Bourbonnais , M. G. Paice , FEBS Lett. 1990, 267, 99. 2365094 10.1016/0014-5793(90)80298-w

[140] C. W. J. Murnaghan , W. G. Forsythe , J. H. Lafferty , G. N. Sheldrake , Green Chem. 2024, 26, 10851. 39474236 10.1039/d4gc01720j PMC11516409

[141] H. Zhang , R. Liu , Y. Xin , Y. Li , G. Shi , R. Zhu , L. Zhang , ACS Sustainable Chem. Eng. 2024, 12, 5842.

